# The effect of royal jelly and tocotrienol-rich fraction along with calorie restriction on hypothalamic endoplasmic reticulum stress and adipose tissue inflammation in diet-induced obese rats

**DOI:** 10.1186/s13104-020-05258-0

**Published:** 2020-08-31

**Authors:** Pardis Irandoost, Naimeh Mesri Alamdari, Atoosa Saidpour, Farzad Shidfar, Farnaz Farsi, Mohammad Asghari Jafarabadi, Mohammad Reza Alivand, Mohammadreza Vafa

**Affiliations:** 1 Student Research Committee, Department of Nutrition, School of Public Health, Iran University of Medical Sciences, Tehran, Iran; 2 National Nutrition and Food Technology Research Institute, Department of Clinical Nutrition and Dietetics, Faculty of Nutrition Sciences and Food Technology, Shahid Beheshti University of Medical Sciences, Tehran, Iran; 3 Department of Nutrition, School of Public Health, Iran University of Medical Sciences, Tehran, Iran; 4 Colorectal Research Center, Iran University of Medical Sciences, Tehran, Iran; 5 Road Traffic Injury Prevention Research Center, School of Health, Tabriz University of Medical Sciences, Tabriz, Iran; 6 Department of Medical Genetics, Faculty of Medicine, Tabriz University of Medical Sciences, Tabriz, Iran

**Keywords:** Obesity, Endoplasmic reticulum stress, Inflammation, Royal jelly, Tocotrienol-rich fraction

## Abstract

**Objectives:**

Endoplasmic reticulum (ER) stress causes adipose tissue dysfunction and chronic inflammation in obesity. Royal jelly (RJ) and tocotrienol-rich fraction (TRF) are reported to ameliorate inflammation. However, the improving effects of RJ and TRF on inflammation from ER stress modulating view have not been assessed so far. Hence, we investigated the effect of RJ and TRF on ER stress and some adipose tissue-derived inflammatory markers in the high-fat diet (HFD)-induced obesity. Wistar obese rats randomly allocated into 5 groups: HFD, calorie restriction diet (CRD), RJ + CRD, TRF + CRD, RJ + TRF + CRD. After 8-week intervention, adipose tissues and hypothalamus were dissected and serum was collected.

**Results:**

RJ reduced glucose-regulated protein-78 (GRP78) expression as ER stress indicator in WAT and hypothalamus compared to CRD. Besides, RJ diminished the expression of inflammatory markers in white adipose tissue (WAT) and also decreased the serum concentration of them. TRF reduced inflammatory markers in the serum without remarkable effects on ER stress. Overall, RJ has protective effect against adipose tissue dysfunction and inflammation then suggested as a therapeutic approach to reduce some obesity-related complications. The impact of TRF in this regard is lower than RJ and limited to systemic inflammation improvement without remarkable changes in adipose tissue inflammation.

## Introduction

Obesity is one of the major causes of chronic disease worldwide and its association with inflammation is well established [1]. White adipose tissue (WAT) regulates body metabolism by releasing several hormone and cytokines [2, 3]. High amount secretion of inflammatory cytokines from expanded WAT and disproportion in pro-inflammatory and anti-inflammatory biomarkers such as tumor necrosis factor-alpha (TNF-α), monocyte chemoattractant protein 1 (MCP1), interleukin-6 (IL-6) vs interleukin-10 (IL-10), adiponectin contribute to obesity-related complications [4, 5].

Several obesity-induced disorders are etiologically attributed to chronic inflammation in relation to obesity [6, 7]. Increasing evidence points to another inflammatory state called endoplasmic reticulum (ER) stress that is a condition occurs with nutritional excess in obesity and metabolic dysfunction [8, 9]. Collected investigations reported the noticeable activation of ER stress through high-fat diet (HFD)-induced obesity [8, 10]. Also, a growing body of evidence has demonstrated the interaction between ER stress and the pathology of obesity [11–13]. The ER is a vital intracellular organelle that organizes the synthesis, folding, and loading of the proteins [14]. Inconsistency between that function due to cellular stress conditions, cause unfolded protein response (UPR) and ER stress [14, 15]. Glucose-regulated protein-78 (GRP78) because of its role in protein folding consider as a key regulator of ER stress [9]. ER stress in adipose tissue reduces thermogenesis and plays an important role in adipose tissue dysfunction, changing cytokine secretion, and causing chronic inflammation in adipocytes [3, 14]. Besides, improving hypothalamic ER stress triggers WAT browning, which in turn diminish inflammatory factors secretion from adipocytes, and finally ameliorate obesity-related disorders [11].

Nowadays, regarding inflammation consequences, there is a potential interest to reverse the obesity-induced ER stress and inflammation. In this regard, Gregor et al. showed that weight loss reduced ER stress and GRP78 in obesity [9]. Moreover, a growing body of evidence has focused on functional foods with anti-inflammatory properties [15, 16].

Royal jelly (RJ) is an important product of honey bees with anti-oxidant and anti-inflammatory properties [17–19]. In addition, anti-inflammatory activities are proposed for tocotrienols (T3), an important part of vitamin E family [20–22]. Kim et al. study demonstrated the effect of T3 on hepatic inflammation by modulating ER stress [16]. However, up to the best of our knowledge, the potential inhibitory impacts of RJ and T3 on ER stress in the hypothalamus, as a regulatory tissue, and adipose tissues, as important sites balancing cytokine secretion, have not been assessed yet. Accordingly, this study aimed to examine the effect of RJ and T3 through calorie restriction diet (CRD) on WAT, brown adipose tissue (BAT), and hypothalamic ER stress and some inflammatory markers in HFD-induced obesity in the rat model.

## Main text

### Materials and methods

#### Animals and experimental design

Fifty-five male Wistar rats were purchased from the Pasteur Institute (Tehran, Iran) at 4 weeks of age and weight of 55 ± 4 g then animals were individually kept in cages then acclimatized under controlled conditions (rooms with 21–24 °C, 50–60% relative humidity and12/12 h reverse light–dark cycle) for 1 week. All procedures handling and using animals have been approved by the Ethics Committee of the Iran University of medical sciences and were based on National Institutes of Health guide for the care and use of laboratory animals [23].

First, rats were randomly placed on the ad libitum HFD group (n = 50) comprising 60% kcal from fat and normal diet (ND) group which fed standard laboratory chow diet (n = 5) for 17 weeks. The animals were weighed every week. When the mean weight of HFD-fed rats became significantly more than rats in the ND group and obesity model induced, rats were randomly divided into one of the following five groups for an additional 8 weeks (n = 10/group): I. RJ fed during CRD II. TRF fed during CRD III. RJ + TRF fed during CRD IV. CRD with no supplementation and V. HFD with no supplementation (remain on HFD). The sample size determined according to previous similar studies [24].

The composition of CRD was similar to HFD with 30% calorie restriction (Appendix 1). RJ group was treated with 100 mg/kg lyophilized RJ powder containing 6% of trans-10-hydroxy-2-decenoic acid and TRF group was treated with 85 mg/kg TRF. The TRF is composed of 11.9% (w/w) α-tocopherol, 12% α-tocotrienol, 2% β-tocotrienol, 19.3% γ-tocotrienol, 5.5% δ-tocotrienol, and 23.5% α-tocopherol. Administered doses of RJ and TRF were chosen considering previous studies based on no observed adverse effects [25, 26].

Finally, animals were subjected to 12 h fasting and anesthetized with an injection of xylazine (xylazine 2%, 20 mg ml−1, Alfasan, Netherlands), and ketamine (ketamine 10%, 100 mg ml−1, Alfasan, Netherlands) intraperitoneally, then blood was collected via cardiac puncture. Blood samples were centrifuged at 2500×*g* for 15 min and serum samples were frozen at − 80 °C. The inguinal WAT, interscapular BAT, and hypothalamus were removed, washed with phosphate-buffered saline (PBS), and were frozen at −80 °C in RNAlater stabilization solution until gene expression analyses.

### Biochemical assay

The concentrations of TNF-α and MCP-1 in serum were measured using Rat TNF-α and MCP-1 Immunoassay kit (MyBioSource, Inc., San Diego, USA) according to the manufacturer’s protocols.

### RNA isolation and quantitative real-time PCR

Total RNA of the WAT, BAT, and hypothalamus was extracted with Trizol Reagent. Synthesized complementary DNA (cDNA) via reverse transcription was amplified by real-time PCR on a quantitative PCR System. PCR amplification was conducted with a fluorescence thermal cycler system using SYBR green kit and rat specific primer sequences for GRP78, TNF-α, and MCP-1 as target genes and β-actin as the housekeeping gene. All of the mentioned experiments were performed with experts who were blinded to treatment groups.

### Statistical analysis

All data were expressed as the mean ± SEM. t test and one-way analysis of variance (ANOVA), followed by Tukey’s post hoc test performed to compare the difference between two and more than two groups respectively. *P*-value < 0.05 was considered statistically significant. The Prism software, version 6·0 (GraphPad, CA, USA) was used for drawing figures.

## Results

We didn’t miss any animals and analysis were done on all of the included rats. Obese animals following CRD remarkably reduced weight relative to HFD. When CRD-fed rats treated with RJ and RJ + TRF significantly more weight loss occurred compared to CRD alone. TRF couldn’t diminish more weight than CRD did (Table 1).

**Table 1:** Comparison of weight and inflammatory indices in interventional groups

Variables	RJ	TRF	RJ + TRF	CRD	HFD
Weight	371.53 ± 8.22	404.74 ± 10.62	369.81 ± 6.58	404.26 ± 8.65	493.28 ± 8.23
P-Value*	0.045	0.989	0.032	< 0.001**	
Weight change	− 67.21 ± 4.84	− 44.40 ± 3.36	− 73.29 ± 4.52	− 40.70 ± 6.50	+ 37.04 ± 5.56
P-value*	0.003	0.955	< 0.001	< 0.001**	
TNF-α (pg/ml)	60 ± 2.63	56.84 ± 3.24	58.75 ± 4.78	79.66 ± 6.40	100.01 ± 6.09
P-value*	0.001	0.002	0.003	0.034**	
MCP1 (pg/ml)	77.29 ± 2.10	82.45 ± 3.9	72.19 ± 2.94	99.41 ± 4.96	131.12 ± 7.37
P-value*	< 0.001	0.009	< 0.001	0.002**	

Data are shown as the mean ± SEM; RJ, royal jelly; TRF, tocotrienol rich fraction; CRD, calorie restriction diet; HFD, high fat diet; TNF-α, tumor necrosis factor-α; MCP1, monocyte chemotactic protein1. * P-Value is indicated RJ, TRF and RJ + TRF versus CRD group by one-way ANOVA; ** P-Value is indicated CRD versus HFD group by t-test

**Fig. 1:**
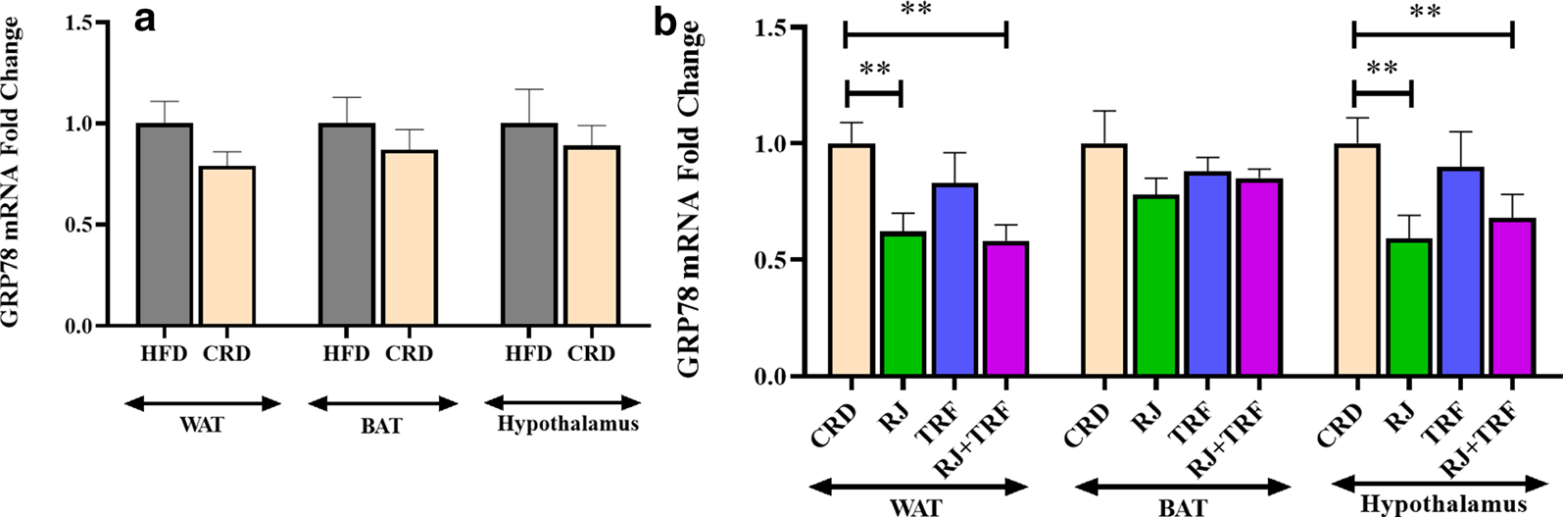
**a** GRP78 mRNA fold change in CRD (n = 10) vs HFD (n = 10) in WAT, BAT, hypothalamus; **b** GRP78 mRNA fold change in RJ (n = 10), TRF (n = 10) and RJ + TRF (n = 10) in WAT, BAT, hypothalamus vs CRD. Data shown as mean ± SEM; **P < 0.05 versus control

We evaluated ER stress in WAT, BAT, and hypothalamus by measuring expression of GRP78 following CRD alone and together with RJ and TRF. CRD reduced ER stress in the hypothalamus, WAT, and BAT but the changes in mRNA levels of GRP78 did not reach significant levels (Fig. 1a). When RJ was added to CRD, the mRNA level of GRP78 in WAT and hypothalamus, but not in BAT, significantly decreased in RJ and RJ + TRF groups relative to the CRD group. However, TRF could not decrease GRP78 at significant levels in assessed tissue in comparison to the CRD group (Fig. 1b).

**Fig. 2:**
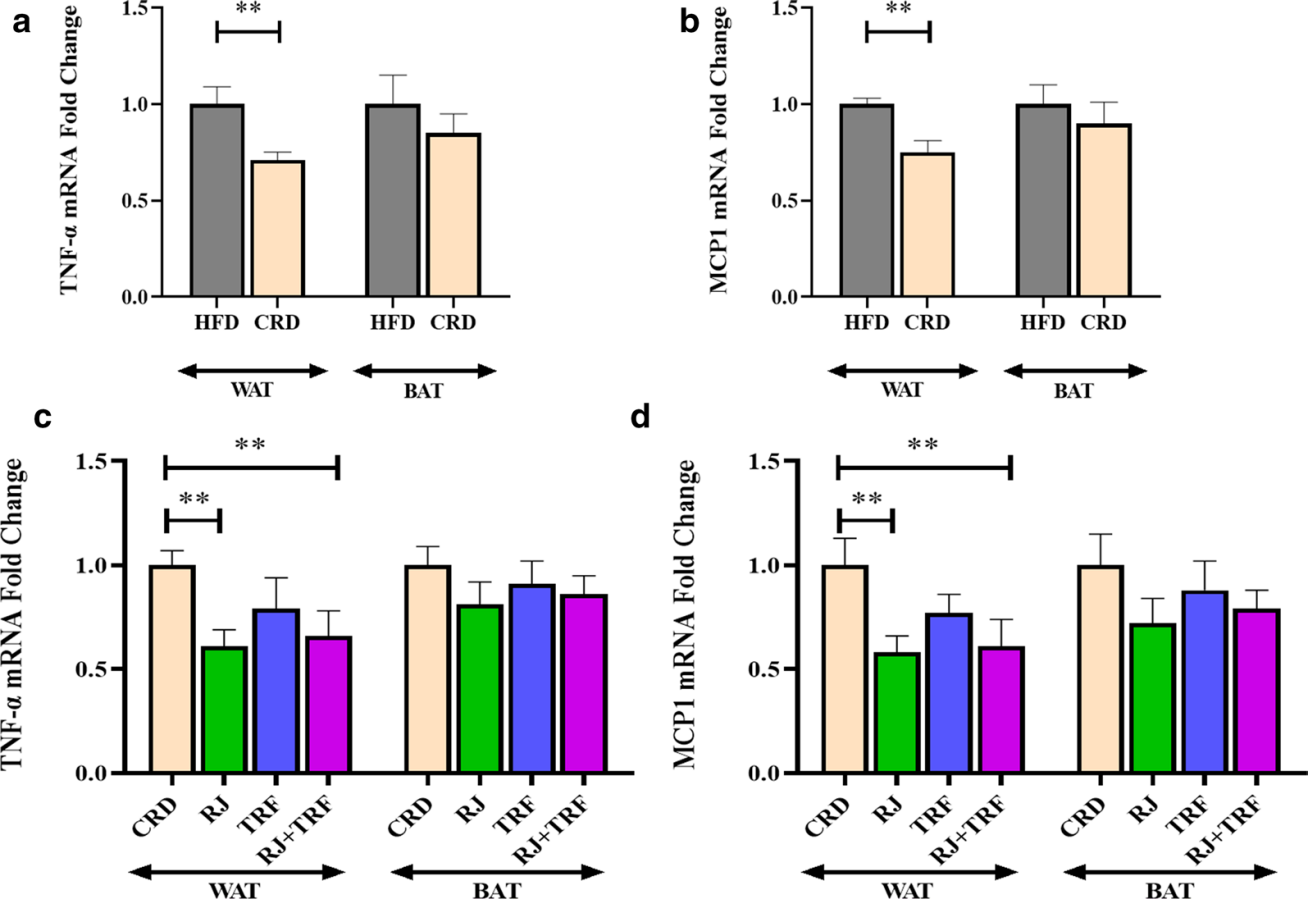
mRNA fold change in CRD (n = 10) vs HFD (n = 10) in WAT and BAT for (**a**) **TNF-** α; (**b**) MCP1. mRNA fold change in RJ (n = 10), TRF (n = 10) and RJ + TRF (n = 10) in WAT and BAT vs CRD for (**c**) **TNF-** α; (**d**) MCP1. Data shown as mean ± SEM; **P < 0.05 versus control

Furthermore, we assessed inflammation in WAT and BAT via measuring the expression of TNF-α, MCP1 and their serum concentration. The expression of TNF-α and MCP1 was diminished in WAT of CRD-fed rats relative to the HFD group (Fig. 2a, b). Their concentration in serum also remarkably reduced following CRD (Table 1). As shown in Fig. 2c and d, after supplementation with RJ and RJ + TRF the mRNA level of TNF-α and MCP1 in WAT decreased remarkably compared to CRD, however TRF did not. The changes of TNF-α and MCP1 expression in BAT neither in RJ nor in TRF groups reach significant levels. Furthermore, the serum concentrations of TNF-α and MCP1 reduced significantly after treatment with RJ, RJ + TRF and TRF (Table 1).

## Discussion

Emerging evidence indicates that several obesity-related disorders etiologically resulted from imbalanced inflammatory markers [6, 7]. Furthermore, the available evidence demonstrates the role of hypothalamic ER stress in the pathogenesis of HFD-induced obesity in the animal model. It is revealed that hypothalamic ER stress causes adipose tissue dysfunction and consequently more inflammatory cytokine secretion [11, 27].

In the current experiment, we realized 8-weeks CRD reduced GRP78 in the hypothalamus and both WAT and BAT of obese rats but not significantly. The magnitude of the CRD effect on ER stress reduction in a previous study was remarkable than in our investigation, possibly because of far greater weight loss relative to our study (40% vs 9.14%) [9]. Also, the high-fat percentage of CRD consumed in our study may have reduced the effect of calorie restriction on ER stress improvement. We also measured systemic inflammation by assaying TNF-α and MCP1 which diminished with CRD compared to HFD. Furthermore, the expression of TNF-α and MCP1 in WAT decreased significantly. Obtained results indicated that CRD could diminish the inflammatory markers in adipocytes but the composition of diet along with calorie restriction is an important determinant in ER stress. When we added RJ to CRD, the expression of GRP78 was reduced and the ER stress was suppressed in both WAT and hypothalamus. Moreover, RJ exerted a suppressive effect on inflammatory parameters in adipose tissue and serum so caused a noticeable reduction in inflammation compared to the CRD alone. To the best of our knowledge, no study has examined the anti-inflammatory effects of RJ from ER stress modulating view. Significant weight loss in the RJ group in our study compared to the CRD group could be a contributing factor in ER stress decrement. In the recent investigation, RJ could not reduce the ER stress dramatically in BAT. It is suggested that obesity-related ER stress is more evident in WAT than in BAT [28]. Perhaps, during higher ER stress, RJ exerts more significant effects.

TRF, neither in adipose tissue nor in the hypothalamus, significantly decreased the mRNA levels of GRP78 more than CRD did in obese rats. However, TRF consumption reduced TNF- and MCP-1 in serum more than CRD, but not in adipose tissues. Subsequently, TRF could alleviate some obesity-related complication such as vascular problems. In recent study, systemic inflammation decrement is independent of ER stress reduction in inguinal WAT and hypothalamus. Our finding demonstrated inflammation reduced in the inguinal WAT of rat-fed TRF but didn’t reach significant levels. Perhaps, TRF caused more inflammation reduction in visceral WAT and other tissues. Our results are in line with previous reports of the anti-inflammatory effect of TRF but do not confirm some previous studies about the inhibitory effect of γT3 on ER stress [16, 29]. These studies evaluated the effect of pure γT3 on hepatic ER stress which possibly exerts stronger effects than TRF does. The inhibitory effects of α-tocopherol against T3 and their possible interaction cannot be ignored [30].

Contreras et al. showed that ER stress suppression in the hypothalamus triggered WAT remodeling and BAT activation via inducing the sympathetic nervous system (SNS) [11]. Besides, we recently revealed that RJ administration activated BAT and also caused WAT remodeling through sympathetic activation [31]. Therefore, we suggest that RJ has a critical role in the modulation of hypothalamic ER stress and leads to SNS activation, which in turn activates WAT browning and decreases ER stress in WAT and attenuates obesity-induced inflammation.

RJ + TRF group demonstrated anti-inflammatory effects similar to RJ-fed rats. regarding the minor effect of TRF in ER stress suppression, it is therefore likely that the RJ is responsible for ER stress modulation in the RJ + TRF group.

Overall, the current study implies that RJ along with CRD protects against HFD-induced ER stress in the hypothalamus and WAT. We suggest that ER stress modulation seems to be a possible mechanism by which RJ exerts some of its anti-inflammatory properties then improve adipose tissue dysfunction and inflammatory condition in obesity more than CRD alone. Hence, RJ could be considered as a therapeutic approach to diminish some obesity-related disorders.

## Limitation

The most important advantage of the recent investigation was that this study was the first one assessed the effect of ER stress modulatory effects of RJ and TRF in the obesity model. The main limitation of this study was we don’t use genetically modified rats. Therefore, our findings need to be confirmed with more study in this regard with knockout models for evaluating the role of ER stress and GRP78 in the future.

## Data Availability

The datasets were used in current study are available from the corresponding author on reasonable request.

## Appendix 1

See Table 2.

**Table 2:** Composition of consumed diets

Dietary composition (g/kg)	ND	HFD
Carbohydrate	536.2	335.125
Fiber	42	26.25
Protein	260.8	163
Lipid	40	400
Calcium	9.5	5.93
Phosphorus	6.5	4.06
Salt	5	3.125
Moisture	50	31.25
Ash	50	31.25
Energy density (kcal/g)	3.6	5.6

ND, normal diet; HFD, high fat diet; CRD, calorie restriction diet

## Abbreviations

TNF-α: Tumor necrosis factor-alpha
MCP1: Monocyte chemoattractant protein 1
IL-6: Interleukin-6
IL-10: Interleukin-10
ER: Endoplasmic reticulum
UPR: Unfolded protein response
GRP78: Glucose-regulated protein-78
SNS: Sympathetic nervous system
WAT: White adipose tissue
BAT: Brown adipose tissue
RJ: Royal jelly
TRF: Tocotrienol rich fraction
CRD: Calorie restriction diet
HFD: High-fat diet
HDEA: Trans-10-hydroxy-2-decenoic acid
PBS: Phosphate‐buffered saline
RT-PCR: Real-time reverse transcription polymerase chain reaction
ANOVA: Analysis of variance
